# Enhancing healthspan with Ashwagandha *(Withania somnifera)*: a comprehensive review of its multifaceted geroprotective benefits

**DOI:** 10.1007/s10522-025-10320-0

**Published:** 2025-09-08

**Authors:** Maanya Vittal, Manlio Vinciguerra

**Affiliations:** 1 School of Pharmacy and Biomolecular Sciences, Liverpool John Moores University, Liverpool, UK; 2 Department of Stem Cell Biology, Research Institute, Medical University, Varna, Bulgaria

**Keywords:** Ashwagandha, Healthspan, Aging, Adaptogen, Neuroprotection, Inflammaging

## Abstract

Ashwagandha (*Withania somnifera*), a revered herb in Ayurvedic medicine, has gained significant scientific recognition for its potential to promote healthy aging. Traditionally used as a *Rasayana* or rejuvenator, this potent adaptogen helps the body manage stress and enhance vitality. This review synthesises extensive evidence for its multifaceted anti-aging capabilities, which target key hallmarks of the aging process. The mechanisms underpinning its effects include enhancing telomerase activity to support cellular longevity, combating systemic oxidative stress, and powerfully countering inflammaging by modulating immune responses and lowering inflammatory markers like C-reactive protein. Robust clinical evidence demonstrates its efficacy in improving crucial physiological parameters, including significant gains in muscle strength and size, enhanced cardiorespiratory fitness, hormonal balance, skin health, and improved sleep quality in older adults. Furthermore, trials have consistently shown its ability to improve cognitive function, including memory and information-processing speed, particularly in adults with mild cognitive impairment. Promising preclinical data also highlight its neuroprotective potential in models of Alzheimer’s and Parkinson’s disease. Here, we review the current evidence supports Ashwagandha’s therapeutic potential in extending healthspan and enhancing quality of life. Large-scale, long-term clinical trials using standardized extracts are essential to fully confirm its role in healthy aging within the global population.

## Introduction: the global challenge of an aging population

The world is undergoing a demographic transformation of unprecedented scale and speed, a phenomenon often termed the “age quake,” which is fundamentally reshaping societies and public health landscapes. This historic shift marks the first time that older individuals are beginning to outnumber the young. Projections from the United Nations are stark, indicating that the proportion of the global population aged 65 and over is expected to surge from 1 in 11 in 2019 to 1 in 6 by 2050 (“Ageing population | Centre for Ageing Better,” n.d.). The most rapidly growing segment is the oldest old; the number of persons aged 80 or over is projected to triple, from 143 million in 2019 to 426 million by 2050. This demographic evolution, driven by decades of falling fertility rates and remarkable increases in life expectancy, represents both a triumph of modern healthcare and a profound societal challenge. The resulting pressures on economic structures, social security systems, and healthcare infrastructure compel a global re-evaluation of long-term strategic planning (“An Aging World: 2015,” n.d.).

This celebrated success in extending human lifespan, however, has unveiled a significant paradox: the years gained are often burdened by illness. A longer life does not necessarily equate to a healthier life. The aging process is intrinsically linked to a heightened vulnerability to a host of non-communicable diseases (NCDs), which have become the leading cause of morbidity and mortality worldwide. The Global Burden of Disease studies underscore this, with data showing that cardiovascular diseases, cancers, chronic respiratory diseases, and neurological disorders now represent the primary health burden in the aging population. This has led to an epidemic of multimorbidity, the co-existence of two or more chronic diseases in an individual. Foundational research from a cross-sectional study of 1.75 million patients revealed that while only 23% of the total population had multimorbidity, this figure rose dramatically to 65% in those aged 65–84 and soared to over 82% in those aged 85 and older (Barnett et al. 2012).

This high prevalence of concurrent chronic conditions creates a cascade of negative consequences. It diminishes the quality of life for millions, leads to complex clinical management challenges including polypharmacy, and places immense, often unsustainable, strain on healthcare systems globally. The underlying biological driver of this increased vulnerability is the aging process itself, a gradual accumulation of damage across multiple systems. Scientists have categorized the fundamental drivers into interconnected hallmarks of aging, such as cellular senescence, telomere attrition, mitochondrial dysfunction, and chronic, low-grade inflammation, termed “inflammaging” (Widodo et al. 2009), which seems to be more typical of the industrialized world (Franck et al. 2025). This recognition has catalysed a critical shift in perspective. To effectively address the health challenges of the twenty-first century, medicine must pivot from a reactive model of treating individual diseases to a proactive framework focused on promoting healthy aging and extending healthspan*—*the period of life spent free from chronic disease and disability (Yadav et al. 2025)(Longo et al. 2015).

This new paradigm necessitates the discovery and validation of safe, effective, and accessible geroprotective interventions that can target the root causes of aging. In this context, there is a burgeoning scientific and public interest in the pharmacopoeia of traditional medicine systems, which have focused on promoting vitality and longevity for millennia (Kaeberlein 2017). Among the most promising of these is *Withania somnifera*, a botanical more commonly known as Ashwagandha (Salve et al. 2019). Within Ayurveda, the traditional medicine system of India, Ashwagandha holds a place of honour. It is a cornerstone of this tradition and is revered as a *Rasayana*, a special classification for herbs and formulations prized for their ability to rejuvenate the body, nourish tissues, promote longevity, and sustain vitality (Koval et al. 2021). The name Ashwagandha itself translates from Sanskrit to “the smell of a horse,” alluding to the traditional belief that it imparts the strength and stamina of a stallion. For centuries, it has been prescribed not to target a single ailment, but to build holistic resilience against physical and mental stress (Bonilla et al. 2021).

This revered status has made Ashwagandha a prime candidate for modern scientific scrutiny, which has sought to understand the molecular basis for its wide-ranging benefits. Contemporary research has positioned it as a premier adaptogen, a class of natural substances that enhance the body’s ability to cope with stressors and maintain physiological equilibrium, or homeostasis (Salve et al., 2019). This adaptogenic quality is profoundly relevant to aging, as the aging process can be defined as a progressive loss of resilience and homeostatic capacity. As the scientific community delves deeper, it is uncovering that the mechanisms of action for this ancient herb overlap significantly with the core biological hallmarks of aging (Priyanka et al. 2020). This review synthesises the current body of scientific evidence to provide a detailed overview of Ashwagandha’s role in promoting healthy aging. By bridging the gap between its traditional use as a *Rasayana* and modern, evidence-based validation of its effects, we aim to provide a comprehensive evaluation of Ashwagandha as a promising therapeutic agent for enhancing healthspan in our rapidly aging global population.

## Ashwagandha: from traditional *Rasayana* to modern adaptogen

Within the vast pharmacopeia of Ayurveda, Ashwagandha holds a place of honour, celebrated for over 3000 years as a powerful healing botanical. As already mentioned, it is best understood through its classification as a *Rasayana*, a term for a select group of plants and formulations prized for their ability to rejuvenate the body, promote longevity, and sustain vitality (Singh et al. 2011). For centuries, Ayurvedic physicians have prescribed it to build resilience against physical and mental stress, enhance cognitive function, and increase overall energy. Its traditional role was not to target a single ailment, but to nourish the body holistically, making it more robust and adaptable.

This revered status as a life-extending *Rasayana* has made Ashwagandha a prime candidate for modern scientific scrutiny. Researchers have sought to understand the molecular basis for its wide-ranging, non-specific benefits, leading to its classification as a premier adaptogen in contemporary herbal medicine (Salve et al. 2019). As an adaptogen, Ashwagandha has been shown to enhance the body’s ability to cope with stressors of all kinds, helping to regulate key physiological systems and maintain a state of equilibrium, or homeostasis. The scientific investigation into its active compounds, primarily withanolides (see next section), has begun to validate its traditional uses, demonstrating antioxidant, anti-inflammatory, and neuroprotective properties (Debnath et al. 2023). This powerful synergy of a rich history of traditional use and a growing body of rigorous scientific evidence makes Ashwagandha a uniquely compelling botanical for promoting health and vitality in the face of aging.

## Core geroprotective (anti-aging) mechanisms of Ashwagandha

**Fig. 1:**
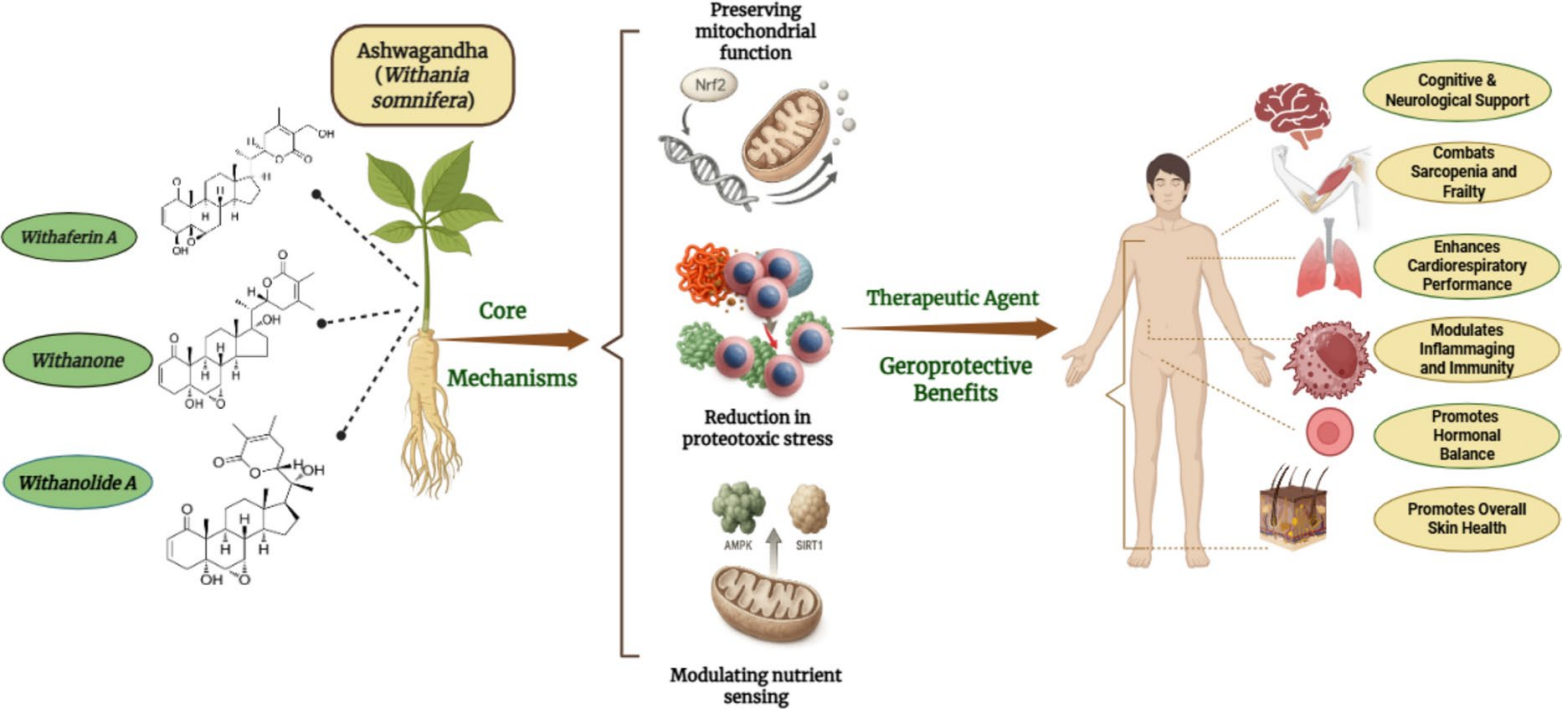
A Visual Overview of Ashwagandha’s Geroprotective Action: From its Bioactive Compounds to Systemic Physiological Benefits and Cellular Rejuvenation

The traditional Ayurvedic classification of Ashwagandha as a *Rasayana* an agent of rejuvenation, is increasingly being validated by modern science, which reveals its ability to intervene directly with the fundamental biology of aging. Its geroprotective potential is not due to a single action but rather a synergistic combination of effects at the molecular level that counter age-related decline (Saleem et al. 2020). This action is driven by a rich diversity of bioactive phytoconstituents, primarily a class of steroidal lactones known as withanolides, including withaferin A, withanone, and withanolide A (Vanden Berghe et al. 2012). These molecules target several of the core, interconnected pathways, or hallmarks, that drive the aging process (Fig. 1) such as:

### Targeting proteostasis and mitigating proteotoxic stress

A central feature of cellular aging is the progressive failure of proteostasis, the complex network of pathways that regulate the synthesis, folding, and degradation of proteins. With age, this quality control system becomes less efficient, leading to the accumulation of misfolded, aggregated, and toxic proteins (Labbadia and Morimoto 2015). This proteotoxic stress is a key pathological feature of many age-related neurodegenerative diseases, such as Alzheimer’s and Parkinson’s disease. Ashwagandha has been shown to directly bolster cellular proteostasis. Its bioactive compounds, particularly withanone, can activate heat-shock proteins (HSPs), which act as molecular chaperones to refold damaged proteins and prevent aggregation (Mikulska et al. 2023). Furthermore, they can enhance the function of the ubiquitin–proteasome system, the cell’s primary machinery for degrading and clearing out toxic proteins, thereby alleviating the burden of cellular damage that accumulates over time (Ghosh et al. 2017).

### *Preserving mitochondrial function *via* the Nrf2 antioxidant pathway*

Mitochondrial dysfunction is another central pillar of aging. As mitochondria, the cell’s powerhouses decline in function, cellular energy production falters, and the output of damaging reactive oxygen species (ROS) increases. This creates a vicious cycle of oxidative stress that damages cellular components, including DNA, lipids, and proteins. Ashwagandha effectively mitigates this age-related mitochondrial decay by upregulating the nuclear factor erythroid 2-related factor 2 (Nrf2) antioxidant defense pathway (Basudkar et al. 2024). Nrf2 is a master regulator of cellular redox balance, and its activation by Ashwagandha’s withanolides triggers the expression of a broad array of protective antioxidant and detoxification enzymes. This action shields mitochondria from oxidative damage, preserves their integrity, and supports robust cellular energy production, thus countering one of the primary drivers of cellular aging (Saleem et al. 2020).

### Modulating nutrient-sensing and longevity pathways

The aging process is intricately controlled by a set of highly conserved nutrient-sensing pathways that coordinate metabolism with cell growth and repair. Key among these is AMP-activated protein kinase (AMPK) and Sirtuin 1 (SIRT1), which are recognized as central regulators of longevity (Wiciński et al. 2024). Interventions known to extend healthspan, such as caloric restriction, exert their benefits largely by activating these pathways. Emerging evidence indicates that Ashwagandha’s influence extends to these critical regulatory hubs. It has been shown to modulate key longevity regulators like AMPK and SIRT1, which in turn orchestrates a pro-longevity program of enhanced cellular repair, improved stress resistance, and reduced inflammation. By engaging these pathways, Ashwagandha can mimic some of the profound cellular benefits of caloric restriction without the need for dietary limitation, positioning it as a potential caloric restriction mimetic (Saha et al. 2024).

By simultaneously addressing proteotoxic stress, metabolic decline, and oxidative damage at a molecular level, Ashwagandha presents a uniquely comprehensive strategy for promoting healthspan. These foundational mechanisms provide a clear biological basis for the wide-ranging physiological, cognitive, and neuroprotective benefits observed in clinical settings, which will be detailed in the subsequent sections.

## Evidence of Ashwagandha and its anti-aging properties

The anti-aging action of Ashwagandha is not due to a single mechanism but rather a synergistic combination of effects at molecular, cellular, and systemic levels. It combats the hallmarks of aging by protecting cells from damage, modulating key genetic and inflammatory pathways, enhancing cognitive and physical function, and bolstering the body’s resilience (Mikulska et al. 2023). In summary, this review illustrates how Ashwagandha exhibits several mechanisms contributing to its anti-aging effects, focusing on five critical areas which are:

### Enhancing physiological parameters and physical performance

The aging process is characterized by a gradual decline in physiological function, including reduced muscle mass and strength (sarcopenia), diminished cardiorespiratory endurance, hormonal imbalances, and poor sleep quality. Research indicates that Ashwagandha can significantly mitigate many of these age-related declines.

Preclinical studies in model organisms provided the foundational evidence for Ashwagandha’s pro-longevity effects. At a fundamental cellular level, a key marker of aging is the shortening of telomeres, the protective caps on our chromosomes. An extract of Ashwagandha root has been shown in vitro to significantly boost the activity of telomerase, the enzyme responsible for maintaining telomere length (Jacczak et al. 2021). In one study using human cell lines, the extract increased telomerase activity by approximately 45%, suggesting a direct mechanism for promoting cellular longevity (Raguraman et al. 2016). In *Caenorhabditis elegans*, an extract of Ashwagandha root was shown to extend mean lifespan by approximately 20%, while also improving late life healthspan by enhancing pharyngeal pumping and locomotor activity (Thakkar et al. 2025). Further research in *Drosophila melanogaster* (fruit fly) demonstrated that withaferin A, a key withanolide, protects against age-related physiological decline, improves stress resistance, and extends lifespan, establishing its geroprotective potential (Koval et al. 2021).

Building on this, human trials have demonstrated tangible benefits. Maintaining muscle mass is critical. A landmark trial found that Ashwagandha (600 mg/day) in adults undergoing resistance training resulted in a significantly greater increase in muscle strength (e.g., a ~ 46 kg increase in bench press 1-rep max vs. ~ 26 kg for placebo) and muscle size (e.g., an 8.6 cm^2^ increase in arm muscle area vs. 5.3 cm^2^ for placebo) (Wankhede et al. 2015). A similar study confirmed these anabolic effects, showing improvements in both upper and lower body strength and a favourable increase in lean body mass (Bonilla et al. 2021). This is complemented by evidence showing a significant reduction in exercise-induced muscle damage, as measured by lower serum creatine kinase levels, suggesting faster recovery (Verma et al. 2025).

Cardiorespiratory fitness (VO2 max) is a strong predictor of longevity. A trial with healthy athletic adults demonstrated that 12 weeks of supplementation (300 mg twice daily) significantly enhanced VO2 max by 13.6% compared to 9.7% in the placebo group and improved self-reported quality of life scores on the WHOQOL-BREF scale (Strasser and Burtscher 2018). Another study corroborated these findings in elite cyclists, who showed a 12% improvement in time-to-exhaustion on a treadmill test after eight weeks (Shenoy et al. 2012).

The herb’s benefits also extend to skin health, a visible marker of aging. In a randomized, double-blind, placebo-controlled clinical trial on healthy adults with photoaged facial skin, the topical application of a lotion containing 8% standardized Ashwagandha root extract for 60 days led to significant improvements. Compared to the placebo, the Ashwagandha group showed greater improvements in skin hydration (20.66% vs 9.5%), elasticity (16.34% vs 3.73%), and a reduction in trans-epidermal water loss. Physician assessments also noted significant reductions in wrinkles and pore size (Narra et al. 2023).

Hormonal balance is also crucial. A systematic review and meta-analysis concluded that Ashwagandha supplementation significantly increased testosterone levels, with a pooled effect size showing an average increase of 145.7 ng/dL in male participants (Lopresti et al. 2019). Specific trials confirmed its efficacy in improving semen parameters (sperm concentration, motility) in infertile men (Mutha et al. 2025) and boosting testosterone by 14.7% and the anti-aging hormone DHEA-S by 18% in overweight, aging males (Lopresti et al. 2019). In perimenopausal women, an 8-week RCT showed that Ashwagandha significantly reduced menopausal symptoms, as measured by the Menopause Rating Scale (MRS), and improved hormonal balance (Gopal et al. 2021). A clinical trial involving healthy women also found significant benefits for female sexual well-being. Participants showed statistically significant improvements across multiple key domains of the Female Sexual Function Index (FSFI), covering both subjective experience and physiological response (Ajgaonkar et al. 2022).

Finally, sleep quality and stress management are foundational to healthy aging. A comprehensive review found Ashwagandha has a significant beneficial effect on overall sleep (Haber et al. 2024). A trial in healthy older adults (65–80 years) showed improved sleep quality, a significant increase in mental alertness upon waking, and better quality of life scores (Kelgane et al. 2020). Its adaptogenic effects are highlighted by its ability to significantly reduce levels of stress hormone cortisol. A key study on chronically stressed adults reported a 27.9% reduction in serum cortisol levels after 60 days of supplementation, which correlated with dramatic reductions in scores on perceived stress scales (Chandrasekhar et al. 2012). This cortisol-modulating effect is a cornerstone of its anti-aging action, as chronically elevated cortisol accelerates cellular aging (Gómez Afonso et al. 2023).

### Improving cognitive function and neuroprotection

Cognitive decline is one of the most feared aspects of aging. Ashwagandha has emerged as a promising nootropic and neuroprotective agent, with a growing body of clinical evidence supporting its efficacy in ameliorating these changes.

A pivotal 8-week RCT in adults with mild cognitive impairment (MCI) found that Ashwagandha root extract (300 mg twice daily) led to significant improvements in both immediate and general memory, as assessed by the Wechsler Memory Scale-III. Sub-scales such as logical memory and verbal paired associates showed marked improvement. Furthermore, participants demonstrated enhanced executive function, sustained attention, and a faster information-processing speed compared to the placebo group (Choudhary et al. 2017). A more recent 12-week trial in adults with MCI further confirmed these findings, showing significant enhancements in working memory and general memory with a standardized extract (Gopukumar et al. 2021). Another RCT highlighted its ability to improve recall memory and reduce error rates in cognitive tasks, underscoring its potential for those with memory complaints (Leonard et al. 2024).

The benefits extend to healthy populations. A trial in healthy individuals found that Ashwagandha improved reaction time and performance on various attention-based tasks, suggesting a role in maintaining peak cognitive performance (Puttaswamy et al. 2025). Its utility has also been explored in conditions with cognitive deficits, such as bipolar disorder, where an 8-week trial showed it improved auditory-verbal working memory, reaction time, and social cognition (Xing et al. 2022). A systematic review concluded that Ashwagandha intake could indeed improve cognitive function across various populations, including the elderly and those with MCI (Ng et al. 2020).

The mechanisms behind these cognitive benefits are multifaceted and are actively elucidated through preclinical research. In a mouse model of scopolamine-induced amnesia, Ashwagandha was shown to reverse memory deficits by inhibiting the enzyme acetylcholinesterase (AChE), thereby increasing acetylcholine levels in the brain, a mechanism like that of many dementia medications (Gautam et al. 2016). Other animal studies have shown that withanolides can promote the regeneration of neurites and the reconstruction of synapses, directly countering the age-related decline in synaptic plasticity (Kuboyama et al. 2005). In a mouse model of cerebral ischemia, Ashwagandha reversed neuronal impairment and cognitive deficits by restoring cerebral blood flow and reducing inflammation (Zhang et al. 1997). Furthermore, it enhances spatial memory by promoting neurogenesis and increasing levels of brain-derived neurotrophic factor (BDNF) in the hippocampus, the brain’s primary memory centre (Er et al. 2025). It also appears to protect against glutamate-induced excitotoxicity, a common pathway of neuronal damage in both acute injury and chronic disease (Kataria et al. 2012).

### Modulating immunity and ‘inflammaging’

Inflammaging, the chronic, low-grade inflammation that accompanies aging, is a primary driver of most age-related diseases. Ashwagandha possesses potent anti-inflammatory and immunomodulatory properties that directly counter this insidious process.

Clinical studies provide direct evidence of its anti-inflammatory action. A foundational study in chronically stressed adults found that Ashwagandha supplementation significantly decreased C-reactive protein (CRP), a key biomarker of systemic inflammation, by over 30% (Guo and Rezaei 2024). A meta-analysis of multiple RCTs later confirmed that Ashwagandha consistently lowers CRP levels (Raut et al. 2024). Another systematic review highlighted its ability to also reduce levels of other pro-inflammatory cytokines like interleukin-6 (IL-6) and tumour necrosis factor-alpha (TNF-α) (Arshad et al. 2025). This is further supported by research showing its ability to modulate oxidative stress, significantly reducing the lipid peroxidation marker malondialdehyde (MDA) while boosting levels of endogenous antioxidants like superoxide dismutase (SOD) (Paul et al. 2021).

Ashwagandha demonstrates efficacy in managing specific inflammatory conditions. In an RCT on patients with knee joint pain, Ashwagandha significantly reduced pain scores on the Visual Analog Scale (VAS) and improved physical function (Ramakanth et al. 2016). Its benefits in rheumatoid arthritis have also been documented, where it helped reduce disease activity scores. Preclinical research using animal models of arthritis has shown it can reduce pro-inflammatory cytokines, suppress cartilage degradation by inhibiting matrix metalloproteinases, and alleviate pain (Samy et al. 2025). Mechanistically, its active compounds, particularly withaferin A, are known to inhibit the master inflammatory transcription factor NF-κB by blocking the activation of its upstream kinase, IKKβ (Heyninck et al. 2014).

In addition to curbing inflammation, Ashwagandha modulates the immune system to counter age-related immune senescence. A landmark research study in healthy adults demonstrated that supplementation significantly increased the activity of Natural Killer (NK) cells, the body’s first line of defence against viral infections and tumours. The study also noted an increase in the activation of T-cell subsets, indicating a broad-spectrum enhancement of adaptive and innate immunity (Alanazi and Elfaki 2023). A more recent trial confirmed these effects, showing a significant increase in CD4 + T helper cells, CD8 + T cytotoxic cells, and NK cells after 30 days of supplementation, bolstering the body’s surveillance capabilities (Mikolai et al. 2009). This dual ability to suppress chronic inflammation while enhancing protective immune responses makes it a powerful tool for managing the immunological challenges of aging (Della Porta et al. 2023).

### Applications for post-viral syndromes (COVID19)

The COVID-19 pandemic introduced novel health challenges, including the management of post-viral syndromes like “long COVID.” Long COVID is defined as “an infection-associated chronic condition that occurs after SARS-CoV-2 infection and is present for at least 3 months as a continuous, relapsing and remitting, or progressive disease state that affects one or more organ systems”. Beyond multiple diagnosable conditions, long COVID symptoms may include shortness of breath, cough, persistent fatigue, post exertional malaise, difficulty concentrating, memory changes, recurring headache, lightheadedness, fast heart rate, sleep disturbance, problems with taste or smell, bloating, constipation, and diarrhea. The established properties of Ashwagandha led researchers to investigate its potential in the contexts of COVID-19 and long COVID.

Initial research on COVID-19 and Ashwagandha focused on computational and in-vitro studies. Molecular docking simulations suggested that withanone, a key compound, could inhibit the SARS-CoV-2 main protease, an enzyme essential for viral replication, by binding to its active site (Kandagalla et al. 2022). These simulations also proposed that Ashwagandha’s components could interfere with the virus’s ability to bind to host cell receptors like ACE2, potentially blocking viral entry (Balkrishna et al. 2021). Another *in-silico* study proposed that Ashwagandha compounds could modulate the inflammatory cytokine storm by inhibiting key signalling pathways like JAK/STAT, which is often dysregulated in severe COVID-19 (Singh et al. 2022).

These promising preclinical findings paved the way for human trials. An early pilot study in patients with mild COVID-19 suggested Ashwagandha as an adjunct therapy could contribute to faster recovery and resolution of symptoms, likely by mitigating the hyper-inflammatory response (Singh et al. 2023). A larger randomized controlled trial investigated it as a prophylactic and found that while it did not significantly reduce the incidence of infection, it was associated with a higher self-reported quality of life during the pandemic period and showed a trend towards promoting faster recovery in those who did get infected (Kulkarni-Munshi et al. 2025).

Regarding long COVID, a large-scale, randomized, placebo-controlled clinical trial, the “APRIL Trial,” was initiated in the UK to formally assess the efficacy of Ashwagandha in promoting recovery (Mallinson et al. 2025). While results from this major trial are pending, the investigation represents a modern application of its traditional use as a restorative tonic. Providing early clinical support, a published case report has already documented significant improvements in fatigue, sleep, and cognitive symptoms in a long COVID patient after supplementation with Ashwagandha, suggesting its therapeutic potential for this complex post-viral condition (Smith et al. 2023).

### Potential role in neurodegenerative diseases

Neurodegenerative diseases, such as Alzheimer’s, Parkinson’s, and Huntington’s, are among the most devastating age-related conditions. Ashwagandha has shown remarkable potential in preclinical models of these diseases, suggesting it could play a role in slowing or halting their progression by targeting multiple pathological pathways.

In Alzheimer’s disease (AD), a groundbreaking study in a mouse model found that an Ashwagandha root extract induced a liver protein (lipoprotein receptor-related protein 1, or LRP1) that facilitated the clearance of amyloid-beta (Aβ) from the brain. This led to a significant reduction in Aβ plaque burden and a reversal of associated behavioural deficits (Sehgal et al. 2012). Another key study showed withanolide derivatives could promote the reconstruction of both pre- and post-synaptic terminals, directly countering the synaptic loss that underlies cognitive decline in AD (Kuboyama et al. 2005). Furthermore, Ashwagandha protects neurons from Aβ-induced toxicity by reducing oxidative stress, preserving mitochondrial function, and inhibiting apoptosis (Tancreda et al. 2025). It has also been shown to inhibit the enzyme acetylcholinesterase, a mechanism like some existing AD drugs (Idrees et al. 2023).

For Parkinson’s disease (PD), which involves the loss of dopamine neurons, Ashwagandha has also shown promise. In a mouse model of PD, treatment with Ashwagandha extract was found to significantly improve motor function, reduce neuroinflammation by modulating microglial activation, and protect dopaminergic neurons from degeneration (RajaSankar et al. 2009). The mechanisms involve the upregulation of key antioxidant pathways like Nrf2, which boosts cellular defences (Kim et al. 2025), and protection against MPTP-induced neurotoxicity by restoring levels of dopamine and its metabolites in the striatum (RajaSankar et al. 2009).

The herb’s neuroprotective effects extend to other conditions. In a mouse model of Huntington’s disease, an inherited neurodegenerative disorder, Ashwagandha extract improved motor performance, reduced the size of toxic protein aggregates (striatal inclusion bodies), and extended survival (Mohd Sairazi and Sirajudeen 2020). In models of cerebral ischemia (stroke), it has protected brain tissue from damage and improved functional recovery by reducing inflammation and apoptosis in the ischemic penumbra (Raghavan and Shah 2015). Studies have also shown its potential in models of chemotherapy-induced cognitive impairment (chemo brain) and in mitigating neuronal damage following traumatic brain injury (Singh et al. 2024). A systematic review concluded that Ashwagandha holds significant therapeutic potential for a range of neurodegenerative diseases, though it emphasized the urgent need for well-designed human clinical trials to translate these robust preclinical findings into effective therapies (Sandhir and Sood 2017).

The extensive body of research synthesised here highlights Ashwagandha’s remarkable potential as a multifaceted agent for promoting healthy aging. From enhancing physical strength and cognitive function to modulating the intricate pathways of inflammation and neurodegeneration, its traditional status as a *Rasayana* is strongly supported by modern scientific evidence. However, significant challenges remain in translating this promise into mainstream clinical practice which are discussed in the next sections.

## Discussion

*Withania somnifera* holds a celebrated and enduring position within the Ayurvedic tradition as a quintessential *Rasayana*, an agent of rejuvenation. The large volume of research examined in this review offers robust scientific support for this historical reputation, revealing Ashwagandha to be a broad-spectrum geroprotective herb that operates on the aging process at systemic, cellular, and molecular scales (Singh et al., 2011). Its true significance, however, is not found in a list of disconnected benefits, but in its profound ability to function as a systems-level intervention. This represents a conceptual departure from the reductionist, single-target approach that has dominated Western pharmacology for decades. Instead of targeting a single receptor or enzyme to treat a specific pathology, Ashwagandha’s strength lies in its capacity to simultaneously and harmoniously modulate the multiple, interconnected pathways that drive aging. This discussion aims to interpret these findings, connecting its molecular actions to its physiological outcomes and reflecting on the possibilities for advancing human healthspan (Bonilla et al. 2021).

The herb’s potent anti-aging capacity is deeply rooted in its power to neutralize inflammaging—the persistent, low-level inflammation and unchecked oxidative damage that are now understood to be primary drivers of the aging process (Saha et al. 2024). While numerous clinical studies confirm a marked reduction in inflammatory biomarkers, the interpretation of these findings goes deeper than simple anti-inflammatory action. This effect, largely attributed to the capacity of withanolides to suppress the primary inflammatory regulator, NF-κB (Krishnaraju et al. 2023), creates a systemic environment that is less hostile to cellular function and more conducive to repair. This is powerfully illustrated by its effects on physical health. The consistent reports of increased muscle development and power in individuals engaged in resistance training (Wankhede et al., 2015) are not merely an isolated anabolic effect but are likely a direct consequence of this improved internal milieu. Chronic inflammation and stress are highly catabolic processes; the stress hormone cortisol actively promotes the breakdown of muscle protein. By significantly lowering cortisol levels (Chandrasekhar et al. 2012) while simultaneously quelling systemic inflammation, Ashwagandha shifts the body’s physiological balance away from tissue breakdown and towards a pro-anabolic, regenerative state. This is augmented by its formidable antioxidant capabilities, which reinforce the body’s innate defences by activating signalling routes such as Nrf2, thus shielding cellular components from progressive damage (Kim et al. 2025). This mitochondrial protection likely underpins the notable improvements seen in cardiovascular endurance (VO2 max), and even the clinically confirmed improvements in skin moisture and firmness can be interpreted as an external manifestation of this enhanced internal cellular wellness (Narra et al. 2023).

This multi-pronged therapeutic strategy is perhaps most evident and compelling in its influence on the central nervous system. The solid clinical results indicating better cognitive performance in people with mild cognitive impairment (MCI) establish it as a valuable nootropic for staving off age-associated mental decline (Choudhary et al. 2017). These clinical benefits are firmly backed by extensive preclinical research that illuminates a sophisticated, multi-front assault on the pathologies of neurodegeneration. In models of Alzheimer’s disease, Ashwagandha does not just target a single aspect of the disease. Instead, it facilitates the removal of toxic amyloid-beta aggregates from the brain, a central feature of AD (Sehgal et al. 2012). Simultaneously, its active withanolides are known to encourage the growth of neurites and the rebuilding of synapses, directly opposing the loss of neural connections that causes cognitive deficits (Kuboyama et al. 2005). This dual capability to both eliminates harmful proteins and actively foster neuronal repair, positions it as an exceptionally promising candidate. Its effectiveness in Parkinson’s models, where it shields dopaminergic neurons and enhances motor control, further highlights its wide-ranging neuro-restorative capacity (RajaSankar et al. 2009).

These findings reinforce the modern understanding of Ashwagandha as a quintessential adaptogen: an agent that enhances resilience by restoring homeostatic balance. Its application in contemporary health crises like long COVID speaks to this adaptability, where it may help alleviate persistent tiredness, mental cloudiness, and other disabling after-effects by regulating the immune system and calming intense inflammation (Smith et al. 2023). However, a critical discussion must also address the nuances and significant gaps in our current understanding. While we know that withanolides are the primary bioactive compounds, the specific contribution of individual molecules to each clinical outcome remains largely undefined. This has profound implications for the standardization of future clinical-grade extracts, a principal difficulty where inconsistent phytochemistry can lead to variable clinical outcomes (Souiad et al. 2020).

Furthermore, while the preclinical evidence for neurodegenerative diseases is spectacular, it creates a very high bar for clinical validation. The dramatic mechanism of amyloid clearance seen in mouse models has yet to be demonstrated in human AD patients, and translating these complex effects into a large-scale RCT remains a major challenge. This raises further questions about optimal dosage and the necessary duration of treatment for chronic conditions, which are currently unknown. Addressing these obstacles is the next frontier for realizing the full promise of this ancient herb (Yadav et al. 2025) and elevating it from a promising supplement to a validated, mainstream therapeutic capable of truly enhancing healthspan.

## Limitations

Based on the above comprehensive analysis of the reviewed literature, several key limitations emerge that might temper the current enthusiasm for Ashwagandha and clearly define the necessary direction for future scientific inquiry. A persistent and primary challenge that undermines the reliability of clinical findings is the lack of standardization across Ashwagandha products (Table 1). The concentration of bioactive compounds, chiefly the withanolides, can differ significantly based on the plant’s origin, the parts used, and extraction methods. For instance, an extract rich in anti-inflammatory withaferin A may be optimal for joint health, while another high in withanone could be more effective for neuroprotection. This leads to variable phytochemical profiles and, consequently, inconsistent clinical results, making it difficult to establish effective, reliable dosing or compare outcomes across studies. Without a well-characterized, stable extract, the journey from promising research to dependable therapeutic guidelines is significantly hindered.

**Table 1:** Variability in Ashwagandha preparations: Implications for research, standardization, and therapeutic efficacy

Supporting reference(s)	Aspect of variation/preparation type	Description & key components	Implication for research & clinical use	Key challenge/consideration for anti-aging applications	Potential impact on global aging demographics
(Saha et al., 2024)	Plant part used (Root vs Leaf)	Root: Most studied, rich in diverse withanolides Leaf: Contains different or varying concentrations of withanolides, notably withaferin A	Different parts may yield distinct phytochemical profiles, leading to varied therapeutic effects Root is traditionally favoured for *Rasayana* properties	Ensuring which part of the plant is optimal for specific anti-aging outcomes, defining the full spectrum profile of each part	Optimizing consistent benefits for a global population where 1 in 6 people will be aged 65 + by 2050
(Souiad et al. 2020)	Extract type (full-spectrum vs standardized)	Full-Spectrum: Contains all active and inactive compounds in their natural ratio Standardized: Processed to contain a specific concentration of a marker compound (e.g., 2.5–5% total withanolides, or high withaferin A/withanone)	Standardized extracts offer predictable dosing and reproducible results, which are crucial for clinical validation full spectrum may offer synergistic entourage effects	Achieving consistency across products in a market with diverse formulations; ensuring key bioactive markers are truly indicative of efficacy	Crucial for providing reliable interventions to counter multimorbidity, which affects up to 82% of those aged 85 +
(Debnath et al., 2023)	Key bioactive compounds (specific withanolides)	Withanone: Linked to proteostasis, neuroprotection, and heat-shock protein activation Withaferin A: Potent anti-inflammatory (NF-κB inhibition) Withanolide A: Associated with neurite regeneration	The specific ratio and concentration of these compounds determine the primary mechanism and targeted benefit (e.g., more anti-inflammatory vs neuroprotective)	Defining optimal ratios of specific withanolides for different age-related conditions; avoiding “cherry-picking” compounds at the expense of overall plant synergy	Enables precision targeting for major NCDs like neurological disorders, a primary health burden in aging populations
(Kelgane et al. 2020)	Cultivation & geographical origin	Environmental factors (soil, climate, altitude) influence the plant’s secondary metabolite production, affecting the final phytochemistry	Variation in geographical origin can lead to different chemical profiles, impacting consistency and efficacy of raw material	Establishing cultivation best practices and quality control for raw materials to ensure consistent bioactive content before extraction	Ensures a consistent global supply of effective raw material for a world where 1 in 6 people will be aged 65 + by 2050
(Nile et al. 2019)	Extraction methods	Different solvents and techniques (e.g., water extraction, alcohol extraction) can selectively yield different compounds from the plant material	The extraction method directly dictates the types and concentrations of withanolides, and other compounds present in the final product	Developing standardized, scalable extraction methods that consistently produce desired phytochemical profiles for specific therapeutic goals	Directly impacts the ability to deliver consistent benefits to the 426 million people projected to be 80 + by 2050
(Wankhede et al., 2015)	Dosage & administration regimen	Varying dosages (e.g., 300 mg once/twice daily, 600 mg daily) and durations (e.g., 8 weeks, 60 days, 12 weeks) are used across studies	Optimal dosage and duration for chronic age-related conditions may differ significantly from short-term stress relief or acute uses	Determining the precise dose–response relationship for long-term anti-aging effects; understanding the necessary duration for sustained benefits	Maximizes healthspan gains for millions, addressing the paradox that gained years are often burdened by illness
(Nile et al. 2019)	Bio-availability & absorption	How well the active compounds are absorbed into the bloodstream and reach target tissues	Differences in formulation (e.g., piperine addition, specific delivery systems) could significantly impact the actual amount of active compound available to the body	Investigating and optimizing formulations to enhance bioavailability, especially for central nervous system target	Essential for ensuring effective action against age-related cognitive decline, a feared aspect of aging
(Souiad et al. 2020)	Impact on drug interactions	The potential for Ashwagandha compounds to interact with conventional pharmaceutical drugs, especially in older, multimorbid patients	Interactions could alter drug metabolism (e.g., via CYP enzymes), increasing or decreasing drug efficacy or toxicity	Thorough pharmacokinetic and pharmacodynamic studies are essential to characterize potential herb-drug interactions for safe integration	Crucial for safe use by the 65% of 65–84-year-olds with multimorbidity who are likely on polypharmacy
(Souiad et al. 2020)	Quality control & contaminants	Ensuring products are free from heavy metals, pesticides, microbial contaminants, and adulterants	Contaminants can negate benefits and pose significant health risks, especially for long-term use in vulnerable populations like the elderly	Implementing stringent quality control measures and regulatory oversight across the supply chain, from cultivation to finished product	Protects the health and trust of a rapidly growing global aging population seeking natural health solutions

There is a pressing need for more large-scale, long-term, and methodologically rigorous human clinical trials to validate the remarkable findings observed in preclinical models. This is especially true for complex, progressive neurodegenerative diseases. For example, groundbreaking studies in mouse models of Alzheimer’s showed that Ashwagandha could induce the clearance of amyloid-beta plaques and reverse behavioural deficits. While spectacular, these disease-modifying effects have yet to be confirmed in human trials. Many of the existing human studies, though promising, are limited by smaller sample sizes or shorter durations, and often focus on symptomatic improvement in conditions like Mild Cognitive Impairment rather than tracking long-term disease progression. With Ashwagandha’s increasing popularity, particularly among older adults who are often on multiple conventional medications, a deeper understanding of its long-term safety profile is vital. Thorough studies on its potential interactions with pharmaceutical drugs are essential to ensure it can be safely incorporated into health management plans. The current body of research has not yet fully characterized these potential herb-drug interactions, which represents a significant knowledge gap and a barrier to its safe integration into conventional healthcare.

## Future prospects and conclusions

Looking forward, the identified limitations naturally delineate several critical avenues for future research that are essential to fully unlock and validate Ashwagandha’s therapeutic potential as a modern agent for promoting healthspan (Table 2). A foundational step for future research is to move beyond using generic extracts and focus on head-to-head comparative trials of different, well-characterized standardized extracts. This approach will help identify which specific phytochemical profiles are most effective for distinct health outcomes, such as whether an extract is superior for anti-inflammatory effects versus neuroprotection. This would allow for the development of targeted, functional extracts optimized for specific therapeutic goals. There is an urgent need to design and execute more rigorous, large-scale, and long-term human clinical trials. To validate the promising preclinical data, particularly for complex neurodegenerative diseases, future studies must be methodologically robust and of sufficient duration to track the progression of chronic conditions. It is crucial that these trials incorporate objective biomarkers such as measuring C-reactive protein for inflammation or telomerase activity for cellular aging to complement patient-reported outcomes and provide concrete evidence of physiological impact. To move beyond general descriptions of its effects, future research should employ advanced omics technologies like genomics and proteomics to precisely map Ashwagandha’s mechanism of action. This will help identify the exact molecular pathways, genes, and protein targets modulated by the herb, such as clarifying how its compounds regulate the inflammatory pathway or promote the reconstruction of synapses. Such detailed mechanistic understanding is vital for its acceptance in evidence-based medicine. A significant opportunity lies in exploring Ashwagandha’s potential as a preventative agent in populations at high risk for age-related diseases. This includes conducting longitudinal studies in individuals with early signs of conditions like cognitive decline or sarcopenia. Furthermore, data from omics studies can help achieve a significant future goal: identifying biomarkers that could predict an individual’s response to Ashwagandha, paving the way for personalized dosing strategies and transforming its traditional use as a *Rasayana* into a targeted, modern therapeutic.

**Table 2:** Unlocking new potential: Ashwagandha’s evolving role and future research directions in addressing age-related health challenges

Supporting reference(s)	Emerging research area/application	Relevance to aging/geroscience	Current evidence/hint (preclinical/in vitro)	Potential future therapeutic niche/impact	Key challenge/knowledge gap
(Saha et al. 2024)	Epigenetic modulation	Age-related epigenetic changes drive gene dysregulation and cellular dysfunction	Ashwagandha components, especially withaferin A, influence gene expression and non-coding RNAs (e.g., miRNAs like miR-25, miR-181c-5p) related to inflammation and aging. It also restored age-induced changes in circadian clock genes (e.g., rBmal1, rPer1, rCry1)	Epigenetic reprogramming for healthy aging; fine-tuning gene expression to reverse age-related decline; chronomodulation for age-related disorders	Precise identification of target genes/epigenetic marks in humans; long-term human studies to confirm sustained epigenetic remodeling
(Widodo et al. 2009)	Stem cell function & senescence	Decline in stem cell regenerative capacity and accumulation of senescent cells (senolytics) are hallmarks of aging	Withanone has shown to decelerate senescence in normal human fibroblasts by decreasing senescence markers (p21), protecting against oxidative damage, and inducing proteasomal activity. Telomerase activity enhancement has been observed in human cell lines	Senolytic therapy; boosting endogenous stem cell pools for tissue repair and regeneration; preventing cellular aging and tissue degeneration	Identifying specific senolytic compounds; clinical trials targeting senescent cell burden; understanding impact on diverse stem cell types in vivo
(Wang et al. 2024)	Gut microbiome modulation	The gut-brain axis and dysbiosis of the gut microbiome are increasingly linked to inflammaging, neurodegeneration, and metabolic decline	Preliminary evidence suggests Ashwagandha may improve gut parameters, modulate short-chain fatty acids (SCFAs), and positively affect gut microbiota composition indirectly by reducing stress and inflammation	Supporting a healthy “longevity microbiome”; mitigating age-related gut dysbiosis; enhancing nutrient absorption and gut-brain axis health	Direct, large-scale human clinical trials on microbiome composition and function; understanding specific microbial changes and their functional impact; optimal dosing for gut health
(Wiciński et al. 2024)	Metabolic syndrome & cardiometabolic health	Age increases risk of metabolic syndrome, type 2 diabetes, and cardiovascular diseases, contributing to reduced healthspan	Studies suggest benefits on blood glucose regulation, significant reductions in total cholesterol, triglycerides, LDL-C, and increase in HDL-C. Its anti-inflammatory and antioxidant effects are also relevant to cardiometabolic health	Prevention/management of age-related metabolic disorders; cardiovascular protection beyond traditional means; reducing risk factors for chronic diseases	Large-scale, long-term human trials on specific cardiometabolic endpoints (e.g., HbA1c, incidence of cardiovascular events); elucidating precise molecular pathways beyond general antioxidant effects
(Kelgane et al. 2020)	Combating frailty in elderly	Frailty is a major geriatric syndrome characterized by increased vulnerability, reduced physiological reserve, and higher risk of adverse health outcomes	Clinical trials have shown improvement in frailty scores (e.g., FAST, 6MWT), quality of life metrics (PSQI, MMSE, SF-12), and reduction in systemic inflammation (e.g., CRP) in older adults	Direct therapeutic intervention for frailty syndrome; improving functional independence, resilience, and quality of life in very old adults; reducing hospitalization rates	Larger cohorts; longer duration studies with objective measures of physical performance and validated frailty scales; identifying specific biomarkers of frailty reversal
(Wankhede et al., 2015)	Sarcopenia prevention/treatment	Age-related loss of muscle mass, strength, and function (sarcopenia) is a primary cause of physical decline in older adults	Human trials demonstrate significantly greater increases in muscle strength (e.g., bench press 1-RM increase of ~ 46 kg) and muscle size (e.g., arm muscle area increase of 8.6 cm^2^) in resistance-trained adults	Direct intervention for sarcopenia; enhancing physical function and mobility in aging populations; improving quality of life and independence	Confirming effects in diverse elderly populations (not just resistance-trained); elucidating specific anabolic pathways beyond general stress reduction (e.g., protein synthesis, satellite cell activity)
(Guo et al. 2023)	Auto-immune disease modulation	Age-related immune dysregulation can contribute to the development or exacerbation of autoimmune conditions	Preclinical research has shown it can reduce pro-inflammatory cytokines and suppress cartilage degradation in animal models of arthritis (an autoimmune/inflammatory condition). It modulates T-cell subsets and NK cells	Adjunctive therapy for age-related autoimmune conditions (e.g., rheumatoid arthritis, lupus); rebalancing immune responses to reduce self-attack	Well-designed human trials in specific autoimmune diseases; identifying precise immunomodulatory targets in different conditions; long-term safety in immunocompromised patients
(Obhrai et al. 2023)	Osteoporosis & bone health	Age-related bone density loss leads to increased fracture risk, impacting mobility and quality of life in older adults	Some traditional uses point to bone health; preliminary animal studies suggest potential anti-osteoporotic effects by modulating bone turnover markers. Its anti-inflammatory action may also indirectly benefit bone health	Prevention of age-related bone loss; adjunctive therapy for osteoporosis; improving bone strength and reducing fracture risk	Direct human clinical trials on bone mineral density and fracture incidence; specific mechanistic studies on osteoblast/osteoclast activity; optimal dosage for bone health
(White et al. 2016)	Ocular health & vision protection	Age-related macular degeneration (AMD) and cataracts are leading causes of vision loss in the elderly. Oxidative stress is a key contributor	Withanolides possess strong antioxidant properties that could protect retinal cells from oxidative damage. Some traditional texts mention its use for eye health	Prevention or slowing progression of age-related ocular diseases; maintaining visual acuity; reducing oxidative stress in the eye	Specific preclinical models of AMD/cataracts; human clinical trials on visual acuity, retinal health biomarkers, and disease progression
(Al-Khayri et al. 2022)	Kidney health & nephroprotection	Age-related decline in kidney function and increased susceptibility to chronic kidney disease (CKD) are significant health burdens	Preclinical studies suggest antioxidant, anti-inflammatory, and anti-apoptotic effects in models of kidney injury (e.g., drug-induced nephrotoxicity, ischemia–reperfusion injury)	Protecting against age-related kidney decline; adjunctive therapy in early CKD; mitigating drug-induced kidney damage in older adults	Human clinical trials on markers of kidney function (e.g., GFR, creatinine, albuminuria); detailed mechanistic studies on specific kidney cell types

In conclusion, the research surveyed solidifies the scientific basis for Ashwagandha’s esteemed role as a rejuvenator. Its therapeutic value appears to stem not from a singular mode of action, but from its integrated effect on diverse facets of biological aging. The herb demonstrates a remarkable capacity to bolster physical health, support convalescence after viral illness, neutralize the chronic, low-grade inflammation that hastens cellular decline, improve cognition and foster neuro-regeneration. This all-encompassing efficacy presents a clear alternative to the standard therapeutic paradigm of targeting individual disease pathways. By enhancing the entire organism’s robustness, Ashwagandha’s evolution from a traditional *Rasayana* to a scientifically backed adaptogen offers a strong model for future wellness strategies, heralding a new chapter in the use of complex botanical agents to extend the human healthspan.

## Data Availability

No datasets were generated or analysed during the current study.
